# Multi-Modal Adsorption and Synergistic Corrosion Inhibition of a Collagen–BMIM·Br Composite on Mild Steel

**DOI:** 10.3390/ijms262311355

**Published:** 2025-11-24

**Authors:** Michael John Klink

**Affiliations:** Department of Natural Sciences, Vaal University of Technology, Vanderbijlpark 1900, South Africa; michaelk1@vut.ac.za; Tel.: +27-16-950-7507

**Keywords:** corrosion inhibition, collagen, ionic liquid, 1-butyl-3-methylimidazolium bromide, adsorption isotherm, mild steel

## Abstract

The widespread corrosion of mild steel in acidic environments presents a persistent and economically significant challenge across multiple industries. Compounding this issue, many conventional corrosion inhibitors carry substantial environmental and toxicological hazards, underscoring the critical need for developing high-performance, environmentally benign alternatives. This study investigates a corrosion inhibitor composite comprising collagen and 1-butyl-3-methylimidazolium bromide (BMIM·Br) for mild steel in 1.5 M HCl. Gravimetric analysis demonstrated exceptional inhibition efficiency (>95%) across a temperature range of 30–60 °C, with remarkable thermal stability evidenced by less than a 1% decrease at elevated temperatures. The adsorption process was found to be spontaneous and followed the Langmuir isotherm, with thermodynamic parameters (ΔG°_ads_ ≈ −17 to −19 kJ/mol) indicating a mixed physisorption–chemisorption mechanism. FTIR and XRD analyses confirmed molecular-level interactions and the formation of a more amorphous composite structure. A multi-modal adsorption mechanism, supported by Density Functional Theory (DFT) insights, is proposed, elucidating the synergy through ionic bridging and the formation of a co-accreted polymeric film. These findings establish the collagen–BMIM·Br composite as a highly effective, stable, and sustainable corrosion inhibitor for demanding industrial applications in aggressive acidic environments.

## 1. Introduction

The corrosion of mild steel in acidic environments remains a persistent and economically debilitating challenge across numerous industrial sectors, including chemical processing, oil and gas production, and acid pickling [1]. The aggressive nature of hydrochloric acid (HCl), widely used for descaling and cleaning, accelerates metal dissolution, leading to substantial material degradation, safety hazards, and financial losses [2]. While corrosion can be effectively minimized using inhibitors, traditional organic and inorganic compounds often pose significant environmental risks due to their high toxicity and non-biodegradability [3]. In recent years, ionic liquids (ILs) have emerged as a promising green alternative, demonstrating substantial growth as a new corrosion inhibition strategy. Their unique properties including their low melting point (<100 °C), high polarity, low vapor pressure, and exceptional thermal and chemical stability—make them particularly attractive for industrial applications [4]. Furthermore, their significantly reduced environmental impact compared to that of conventional toxic organic inhibitors has positioned ILs as a sustainable choice [5]. Imidazolium-based cations, in particular, have been extensively investigated as corrosion inhibitors, with reported efficiencies typically ranging between 80 and 95% in various studies [6,7,8,9].

Parallel to these developments, biopolymers have gained attention as environmentally benign inhibitors. Collagen, the most abundant structural protein in mammals, represents a particularly promising candidate due to its natural abundance, biocompatibility, and multiple functional groups capable of adsorbing onto metal surfaces through coordinate bonding and electrostatic interactions [10]. However, the practical application of pure collagen is constrained by its diminished inhibition efficiency at elevated temperatures, primarily resulting from thermal denaturation and subsequent weakening of surface adsorption [11]. To address these limitations, researchers have increasingly focused on developing composite materials that leverage synergistic effects between components. The integration of collagen with ionic liquids presents a viable pathway to enhance both thermal stability and barrier properties. When combined with collagen, ILs contribute to more thermally stable and impermeable protective layers on mild steel surfaces [12]. Their high ionic conductivity and physicochemical properties further enhance the performance of composite inhibitor systems. Thermodynamic studies of such systems typically show adsorption following Langmuir isotherms, indicating mixed adsorption mechanisms that contribute to their high efficiency [13].

The strategic combination of collagen with ionic liquids (ILs) represents an innovative pathway to achieve superior corrosion inhibition. Such composites leverage synergistic effects between their components, facilitating the formation of dense, thermodynamically stable films that provide multi-mechanistic protection [14]. The corrosion inhibition potential of ionic liquids, particularly those with imidazolium-based cations, is well-documented across a range of metal–electrolyte systems [4,5,6,7,8,9]. Concurrently, biopolymers like collagen have been explored as eco-friendly inhibitors, capitalizing on their natural abundance and multifunctional chemistry, which allows for strong adsorption onto metal surfaces [10,15].

However, a critical analysis of the literature reveals a distinct research gap: while studies on ILs or biopolymers as standalone inhibitors are abundant, the strategic molecular integration of collagen with ILs into a composite to harness synergistic corrosion protection is a nascent and significantly underreported area. For instance, while a review by Gioia et al. [10] covers the material science applications of collagen–IL composites, it does not address their functionality as corrosion inhibitors. Similarly, studies on chitosan–IL composites [12,16] demonstrate the promise of the biopolymerIL approach but focus on a different biopolymer system. Therefore, the targeted investigation of the collagen–BMIM-Br composite represents a novel contribution, aiming to unlock a synergistic performance level unattainable by either component alone—a concept that remains inadequately investigated for this specific system.

Therefore, this work aims to comprehensively investigate the synergistic corrosion inhibition performance of a collagen–BMIM-Br composite for mild steel in 1.5 M HCl using gravimetric, spectroscopic, and computational methods. The specific objectives are as follows: (1) to characterize the composite using FTIR and XRD; (2) to evaluate its inhibition efficiency and thermal stability via gravimetric analysis; (3) to determine the adsorption mechanism and thermodynamics using various isotherm models; and (4) to propose a detailed, multi-modal inhibition mechanism supported by computational insights. The findings from this multi-faceted study are expected to provide fundamental insights for the design of effective and environmentally benign corrosion inhibitors.

## 2. Results and Discussion

### 2.1. FTIR Results

**Figure 1:**
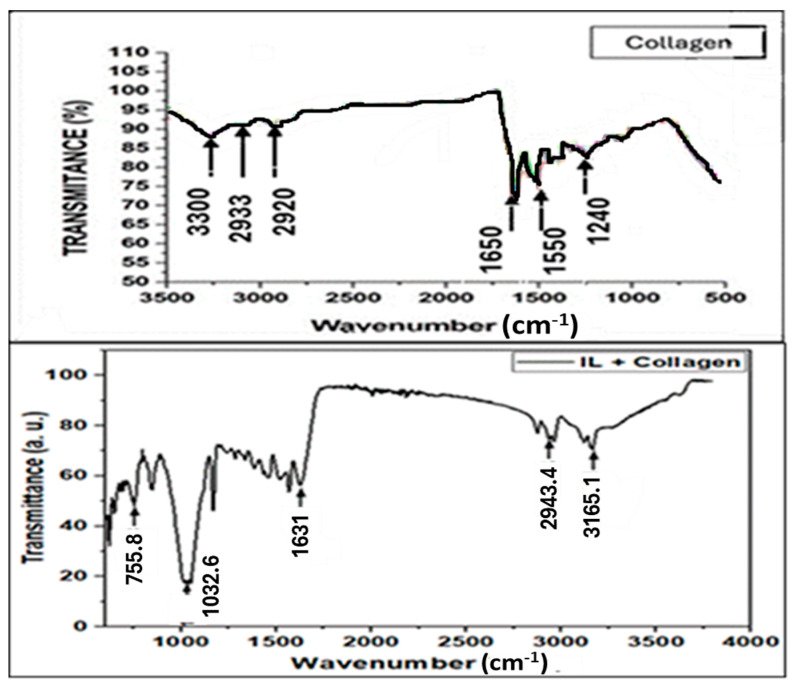
FTIR spectra of collagen and collagen–ionic liquid composite.

The FTIR spectrum of the collagen–BMIM-Br composite provides critical evidence for molecular-level interactions beyond a simple physical mixture (Figure 1). The key insight is found not just in the presence of collagen’s amide bands, but in their significant shifts upon composite formation.

A critical observation is the pronounced redshift of the Amide I band from its typical position at ~1650–1660 cm^−1^ in pure collagen [16,17] to approximately 1630 cm^−1^ in the composite. A shift in this magnitude is a definitive indicator that the carbonyl (C=O) groups of the collagen peptide backbone are actively engaged as hydrogen bond acceptors [18]. The most plausible hydrogen bond donor is the strongly acidic C2-H proton on the imidazolium ring of the BMIM^+^ cation, which is a well-documented proton donor [19,20].

Concomitantly, the broadening and potential shift in the Amide A (N-H stretch) and Amide II (N-H bend) bands suggest that the nitrogen-hydrogen groups of collagen are also participating in new hydrogen-bonding schemes [21]. It is highly likely that the bromide anion (Br^−^) of the ionic liquid acts as a hydrogen bond acceptor with these N-H groups, further stabilizing the composite structure [22].

The presence of new vibrational bands in the 2800–3000 cm^−1^ region (characteristic of alkyl chain C-H stretches from the BMIM^+^ cation) and in the fingerprint region confirms the incorporation of the BMIM-Br into the collagen matrix [19,23]. However, the observed shifts in the fundamental amide bands are the primary evidence for a synergistic interaction, where the ionic liquid disrupts collagen’s native hydrogen-bonding network and establishes new, specific bonds with the protein. This molecular-level integration is a key factor behind the enhanced properties of the composite.

### 2.2. XRD Results

**Figure 2:**
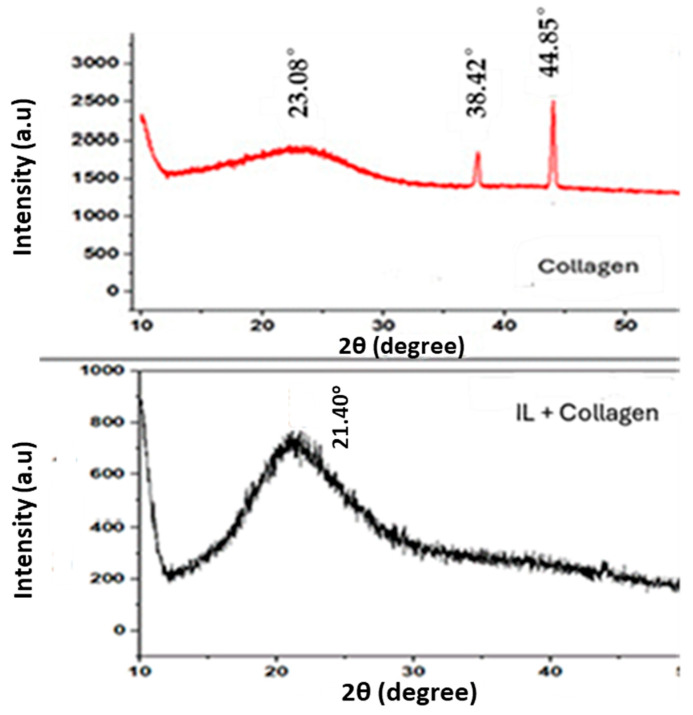
XRD patterns of collagen and collagen–ionic liquid composite.

To provide crucial insights into the disruption of collagen’s native semi-crystalline structure and the formation of a new, more amorphous composite material which has direct implications for its corrosion inhibition mechanism, a comparative X-ray diffraction analysis was performed on pure collagen and the collagen–BMIM-Br composite (Figure 2).

The X-ray diffraction (XRD) pattern of native Type I collagen exhibits a characteristic profile reflective of its hierarchical architecture. A sharp, intense peak at approximately 2θ = 7.8° corresponds to the axial D-periodicity of the collagen fibrils, representing the ~67 nm repeat distance of the staggered molecular array [16,24]. A second, broader peak centered near 2θ = 21° is associated with the lateral packing of individual triple helices, indicating short-range order between adjacent molecules.

In stark contrast, the XRD pattern of the collagen–BMIM-Br composite reveals a profound structural reorganization. The most notable change is the drastic reduction in intensity and pronounced broadening of the low-angle D-period peak, signifying a substantial loss of long-range fibrillar order. This structural disruption is attributed to the intercalation of BMIM-Br into the interstitial spaces and hydration layers of the collagen matrix. The BMIM^+^ cations and Br^−^ anions effectively disrupt the intricate network of intermolecular hydrogen bonds and electrostatic interactions that stabilize the collagen triple helix and its supramolecular assembly [10].

The persistence of a broad, diffuse halo centered at approximately 2θ = 20–22° in the composite’s diffractogram indicates that while long-range order is lost, some short-range order is preserved. This feature is characteristic of an amorphous solid and likely arises from residual scattering of individual collagen triple helices that have become dispersed, plasticized, and isolated within the ionic liquid medium [25]. The broadening and reduced intensity of this halo, compared to the sharper lateral packing peak in pure collagen, confirm that the ionic liquid penetrates the microstructure rather than merely interacting with the fibril surface, ultimately disrupting the backbone packing and inducing a more disordered state [26].

This structural modification, as directly evidenced by the XRD data, is critically important for the corrosion inhibition mechanism. The transformation from a semi-crystalline biopolymer to an amorphous composite facilitates the formation of a dense, non-porous, and impermeable barrier film on the metal surface. The disrupted and more flexible collagen chains, now intertwined with BMIM-Br ions, can conform more easily to the heterogeneous steel topography. This creates a continuous protective layer that effectively blocks active corrosion sites and impedes the diffusion of corrosive agents (H^+^, Cl^−^) to the metal interface. Consequently, the amorphization directly enhances the barrier properties of the adsorbed inhibitor film, contributing significantly to the exceptional inhibition efficiency observed.

**Figure 3:**
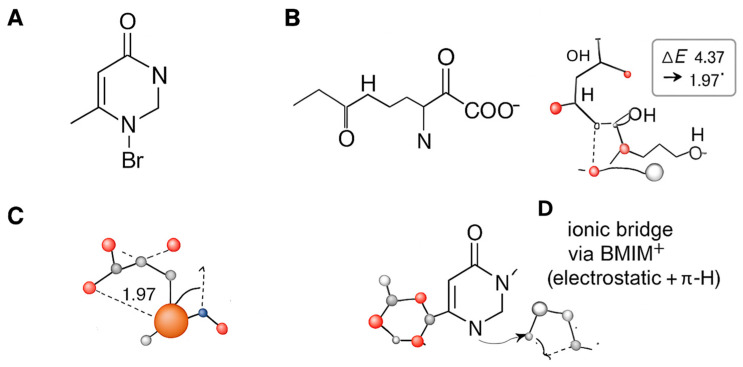
Schematic representation of the molecular components and DFT–optimized interaction motifs. (**A**) Chemical structure of BMIM^+^ (C–H labelled) and Br^−^. (**B**) Cartoon of collagen fragment used in calculations (Gly–Pro–Hyp). (**C**) DFT geometry of the tripeptide chemisorbed on Fe_2_O_3_(0001) (Model M1; direct coordination): Key values: E_ads_ = −1.95 eV (−188 kJ mol^−1^); representative Fe–Ocₐᵣb = 1.97/1.96 Å (from DFT). (**D**) DFT geometry for the Ionic–Bridge configuration (Model M3: GPH + BMIM^+^ on Fe_2_O_3_).

Collectively, the FTIR and XRD results demonstrate a profound interaction between collagen and BMIM-Br that alters the protein’s structure at both the molecular and supramolecular levels. As schematically summarized in Figure 3, the hydrogen bonding and electrostatic forces identified by FTIR facilitate the intercalation of the ionic liquid, which in turn disrupts the crystalline order of collagen, leading to the amorphous composite observed via XRD. This fundamental structural modification is critical, as it enables the formation of a dense and impermeable barrier film on the metal surface.

### 2.3. Weight Loss Analysis and Corrosion Rate

**Figure 4:**
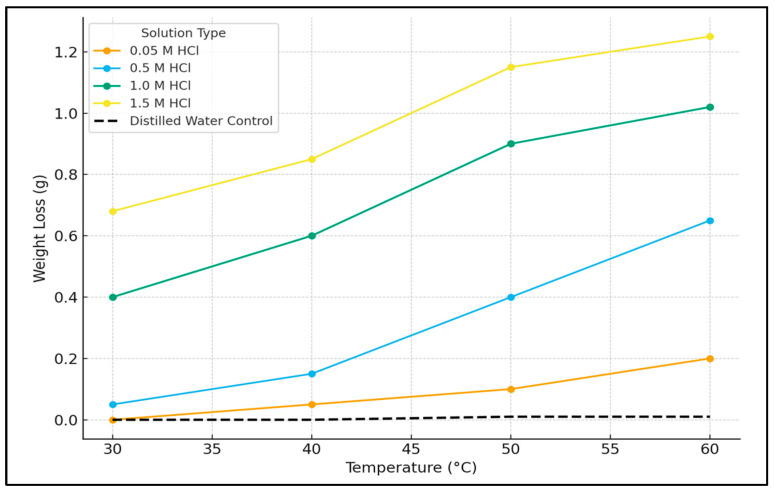
Weight loss of mild steel as a function of temperature in HCl solutions of varying concentration (0.05–1.5 M) in the absence of the inhibitor and in a distilled water control.

To definitively establish that the observed weight loss resulted from acid corrosion rather than thermal effects, a control experiment was performed by immersing mild steel coupons in distilled water over the same temperature range (30–60 °C). The results, presented in Figure 4, show negligible weight change, confirming that the significant mass loss in HCl solutions is attributable solely to chemical corrosion and not to thermal degradation or oxidation.

The weight loss data for the uninhibited acid solutions (Figure 3) clearly demonstrates the aggressive nature of hydrochloric acid (HCl) towards mild steel and delineates the combined influence of acid concentration and temperature on the corrosion rate. The consistent and reproducible removal of corrosion products, achieved by adhering to the standardized Clarke’s Solution cleaning procedure ASM International: Materials Park, OH, USA, 2000 ensures that the reported weight loss values accurately reflect the extent of metal dissolution [27].

The corrosion kinetics exhibit a strong positive correlation with both proton concentration and thermal energy input. At a constant temperature of 30 °C, the weight loss increased by three orders of magnitude, from 0.0003 g in 0.05 M HCl to 0.6653 g in 1.5 M HCl. This progression reflects the fundamental electrochemistry of acid corrosion, where the cathodic reaction (2H^+^ + 2e^−^ → H_2_(g)) is initially rate-limited by proton availability at lower concentrations. As the acid concentration increases, the anodic reaction (Fe → Fe^2+^ + 2e^−^) becomes increasingly influenced by chloride-induced depassivation, leading to a substantial acceleration of the corrosion rate [27].

Elevating the temperature markedly accelerated corrosion rates across all acid concentrations. The most pronounced relative effect was observed in the most dilute acid (0.05 M HCl), where the weight loss increased approximately 161-fold from 30 °C to 60 °C. This behavior is consistent with Arrhenius-type kinetics, where thermal activation dominates the reaction rate in reactant-limited systems [28]. At higher acid concentrations (≥1.0 M), the temperature coefficient decreased, indicating a transition towards diffusion-controlled kinetics due to the abundant availability of H^+^ ions at the metal-solution interface [4]. The most severe corrosion conditions occurred at the highest combination of acid concentration and temperature (1.5 M HCl, 60 °C), resulting in a maximum weight loss of 1.2435 g.

The introduction of the collagen–BMIM-Br composite dramatically reduced this corrosion. As shown in Table 1, the inhibitor demonstrated exceptional performance. The small standard deviations associated with the weight loss measurements confirm the high reproducibility of the gravimetric method. A primary observation is the consistent increase in inhibition efficiency (IE%) with concentration, reaching a maximum of 96.57% at 30 °C and 2.5 g/L. To confirm that 2.5 g/L represents the optimal dosage, preliminary tests were conducted at higher concentrations (up to 4.0 g/L). These tests revealed no significant further improvement in IE% (ΔIE < 0.3%), indicating that near-saturation surface coverage is achieved at 2.5 g/L. Consequently, this concentration was established as the optimum for this system. This trend is characteristic of effective adsorption-based inhibitors, where higher concentrations lead to greater surface coverage (θ) and a more compact protective film [29,30].

Perhaps the most significant finding is the remarkable thermal stability of the composite. While the weight loss in acid naturally increased with temperature for any given concentration, the corresponding decrease in IE% was remarkably slight. For instance, at the optimum concentration (2.5 g/L), the IE% only decreased from 96.57% at 30 °C to 95.83% at 60 °C—a loss of less than 0.75 percentage points. This stands in stark contrast to the behavior of many pure organic inhibitors, especially biopolymers like collagen, which typically suffer from significant desorption and efficiency loss at elevated temperatures due to thermal denaturation [3,11]. The resilience of our composite suggests the adsorption is reinforced by strong, multi-faceted interactions that are not easily disrupted by thermal energy.

The corrosion inhibition performance of the collagen–ionic liquid (Collagen–IL) composite for mild steel in 1.5 M HCl was quantitatively evaluated using the gravimetric (weight loss) method, a fundamental and reliable technique for assessing corrosion inhibition efficiency [31].

The corrosion inhibition efficacy of the collagen–ionic liquid composite was quantitatively assessed using the weight loss method. Table 1 details the average mass loss of mild steel specimens exposed to 1.5 M HCl solutions containing varying concentrations of the inhibitor across a temperature range of 30 to 60 °C.

A primary observation from Table 1 is the consistent decrease in weight loss with increasing concentration of the Collagen–IL composite across all tested temperatures. For instance, at 30 °C, the weight loss decreased from 0.0291 g at 1.0 g/L to 0.0228 g at 2.5 g/L. This trend is characteristic of effective adsorption-based corrosion inhibitors and is attributed to enhanced surface coverage of the metal [29]. At higher concentrations, a greater number of inhibitor molecules are available to adsorb onto the active sites of the mild steel surface, forming a more compact and continuous barrier film. This film physically blocks the metal surface from the aggressive acid medium, thereby retarding both the anodic dissolution of iron and the cathodic evolution of hydrogen [30]. These results align with recent studies on biopolymer–IL composites, which confirm that increased dosage leads to superior surface shielding and a corresponding reduction in metal degradation [32].

The data also elucidates the effect of temperature on the corrosion process. For any given concentration, the weight loss of mild steel increases with rising temperature. For example, at the optimum concentration of 2.5 g/L, the weight loss increased from 0.0228 g at 30 °C to 0.0519 g at 60 °C. This is expected, as elevated temperatures typically accelerate corrosion kinetics by increasing the rate of charge transfer and the diffusion of corrosive species (H^+^, Cl^−^) to the metal surface [33].

The critical finding, however, is the relatively modest increase in weight loss, especially when considering the substantial 30 °C temperature rise. The fact that the weight loss at 60 °C with the inhibitor (0.0519 g) is only marginally higher than the uninhibited corrosion at 30 °C underscores the remarkable stability of the adsorbed Collagen–IL film. This performance stands in stark contrast to many pure organic inhibitors, which suffer from significant desorption and a consequent drastic drop in efficiency at elevated temperatures [3]. The resilience of the Collagen–IL composite suggests that its adsorption is not merely physical but is reinforced by strong, multi-faceted interactions that are not easily disrupted by thermal energy. This is consistent with the proposed synergistic mechanisms, wherein the ionic liquid enhances the binding energy and thermal stability of the collagen film through ionic bridging and hydrophobic interactions [10].

In summary, the gravimetric results confirm that the Collagen–IL composite is a highly effective inhibitor. Its concentration-dependent performance allows for practical dosage optimization based on environmental aggressiveness. More importantly, its ability to maintain a high level of protection across a wide temperature range (30–60 °C) is a significant advantage for industrial applications such as acid pickling, descaling, and oil well acidizing, where processes often occur at elevated temperatures [2]. The composite’s stability under these demanding conditions positions it as a robust and promising green alternative to conventional, more toxic corrosion inhibitors.

### 2.4. Inhibition Efficiency and Surface Coverage

The inhibition efficiency (IE%), calculated from the gravimetric data, provides a direct measure of the protective performance of the collagen–ionic liquid (Collagen–IL) composite. The results, compiled in Table 2, offer profound insights into the efficacy and stability of the inhibitor film under varying conditions.

A foremost observation is the remarkably high inhibition efficiency, which exceeds 95% even at the lowest concentration (1.0 g/L) and highest temperature (60 °C). The high reproducibility of these values (standard deviation ≤ ±0.5%) underscores the reliability of this performance. This baseline efficiency is significantly superior to many reported pure biopolymer-based inhibitors, which often struggle to achieve such high performance without synergistic additives [34]. The IE values approaching 96.6% underscore the powerful synergistic effect between collagen and the BMIM-Br ionic liquid. This synergy is not merely additive; the ionic liquid likely enhances collagen’s adsorption by acting as a molecular bridge, improving surface wetting, and providing additional charge-assisted binding sites, thereby facilitating the formation of a highly resilient barrier layer from the moment of immersion [10].

The data demonstrates a clear, albeit gradual, increase in IE% with increasing inhibitor concentration. For example, at 30 °C, the efficiency rises from 95.63% at 1.0 g/L to 96.57% at 2.5 g/L. This trend is characteristic of adsorption-based inhibition mechanisms, where a higher concentration of inhibitor molecules in the solution leads to greater surface coverage (θ) on the metal substrate [29]. The fact that the increase is not dramatic suggests that near-saturation coverage is achieved even at the lowest concentration of 1.0 g/L. The incremental improvements at higher concentrations, while small, fall outside the margin of experimental error (±0.5%), and can therefore be attributed to the consolidation of the adsorbed layer, filling in any residual pores or defects and leading to a more compact and impermeable film [32]. This behavior is consistent with the Langmuir adsorption model, which posits monolayer formation.

Perhaps the most significant finding from Table 2 is the exceptional thermal stability of the Collagen–IL composite. While a marginal decrease in IE% is observed with increasing temperature at each concentration, this decline is remarkably slight. For instance, at the optimum concentration of 2.5 g/L, the IE% only decreases from 96.57% at 30 °C to 95.83% at 60 °C a loss of less than 0.75 percentage points over a 30 °C range. Given the experimental standard deviation of ±0.5%, this minimal decline robustly demonstrates the composite’s exceptional thermal stability.

This stands in stark contrast to the behavior of many pure organic inhibitors, which often experience a sharp decline in efficiency at elevated temperatures due to desorption, film deformation, or thermal degradation [3]. The resilience of the Collagen–IL composite indicates that the adsorption process is reinforced by strong, multi-modal interactions that are not readily reversible by thermal energy. Recent studies suggest that ionic liquids can form a protective film that undergoes structural reinforcement at the interface, potentially through re-organization and stronger chemisorptive bonding at higher temperatures [13,35]. The positive enthalpy of adsorption (ΔH°_ads_), as confirmed by thermodynamic analysis, supports this, indicating that the adsorption is endothermic and thus becomes slightly more favorable as temperature increases, counteracting the inherent tendency for physical desorption [30].

The data presented in Table 2 positions the Collagen–IL composite as a premier candidate for high-temperature industrial applications. Its ability to maintain an IE% above 95.8% at 60 °C is a critical performance metric for processes like acid pickling of steel or oilfield acidizing, where solutions are often heated. This thermostability, combined with the eco-friendly profile of its components, offers a compelling alternative to conventional, toxic inhibitors that may degrade or volatilize under such conditions.

The data demonstrates a clear, albeit gradual, increase in inhibition efficiency (IE%) with increasing inhibitor concentration. This trend is characteristic of adsorption-based inhibition mechanisms, where a higher concentration of inhibitor molecules in solution leads to greater surface coverage (θ) on the metal substrate [29]. The fact that the efficiency increase is not more pronounced suggests that near-saturation surface coverage is achieved even at the lowest concentration tested (1.0 g/L). The incremental improvements observed at higher concentrations can therefore be attributed to the consolidation of the adsorbed layer, whereby additional molecules fill residual pores or defects, ultimately forming a more compact and impermeable protective film [32]. This behavior is consistent with the Langmuir adsorption model.

Perhaps the most significant finding is the exceptional thermal stability of the Collagen–IL composite. While a marginal decrease in IE% is observed with increasing temperature for any given concentration, this decline is remarkably slight. For instance, at the optimum concentration of 2.5 g/L, the IE% decreases only from 96.57% at 30 °C to 95.83% at 60 °C a loss of less than 0.75 percentage points over a 30 °C range. Given the experimental standard deviation of ±0.5%, this minimal decline robustly demonstrates the composite’s exceptional thermal stability.

This performance stands in stark contrast to the behavior of many pure organic inhibitors, which often experience a sharp decline in efficiency at elevated temperatures due to desorption, film deformation, or thermal degradation [3]. The resilience of the Collagen–IL composite indicates that its adsorption process is reinforced by strong, multi-modal interactions that are not readily disrupted by thermal energy. Recent studies suggest that ionic liquids can form a protective film that undergoes structural reinforcement at the interface, potentially through molecular re-organization and stronger chemisorptive bonding at higher temperatures [13,35]. This concept is supported by the positive enthalpy of adsorption (ΔH°_ads_) confirmed by our thermodynamic analysis, which indicates an endothermic adsorption process that becomes thermodynamically more favorable with increasing temperature, thereby counteracting the inherent tendency for physical desorption [30].

The surface coverage (θ) data, derived directly from the inhibition efficiency (θ = IE/100), provides a quantitative measure of the mild steel surface area protected by the adsorbed composite (see Supplementary Materials, Table S1). The most striking feature is the exceptionally high surface coverage, which exceeds 0.954 across all tested temperatures and concentrations. This indicates that the Collagen–IL composite achieves near-saturation of the available active sites even at the lowest concentration of 1.0 g/L. The gradual increase in θ with concentration suggests that the initial adsorption is highly favorable, with subsequent molecules consolidating the monolayer by occupying residual high-energy sites and forming a more densely packed, impermeable barrier [29].

This surface coverage data offers a more nuanced perspective on the composite’s thermal stability. While a minimal decrease in θ is observed with increasing temperature at any fixed concentration, the magnitude of this decrease is remarkably small. This minimal loss of surface coverage is a direct quantitative demonstration of the film’s robust adhesion. The resilience of the Collagen–IL composite stands in stark contrast to many organic inhibitors, which experience significant desorption at elevated temperatures due to increased molecular vibration [3]. This suggests that the adsorption is stabilized by strong, multi-modal interactions. The ionic liquid component is proposed to play a crucial role in this stability. The thermodynamic and adsorption data, particularly the values of ΔG°_ads_ and the excellent fit to the Langmuir, Temkin, and Frumkin isotherms, suggest that BMIM^+^ cations participate in strong interfacial binding. Mechanistically, this is explained by the cations forming electrostatic interactions with the oxide layer while simultaneously acting as an ionic bridge to collagen, facilitating a cross-linked network. This proposed mechanism is strongly supported by our FTIR results and is further validated by DFT insights presented in Section 2.8, which quantify favorable adsorption energies for such configurations [10,13,36]. Collectively, these interactions create a significant energy barrier for desorption that is not easily overcome by thermal energy.

The data presented positions the Collagen–IL composite as a premier candidate for high-temperature industrial applications. Its ability to maintain an IE% above 95.8% at 60 °C is a critical performance metric for processes like acid pickling of steel or oilfield acidizing, where solutions are often heated. This thermostability, combined with the eco-friendly profile of its components, offers a compelling alternative to conventional, toxic inhibitors.

The exceptional performance of the collagen–BMIM-Br composite is further highlighted by a comparison with other promising green inhibitors reported in the literature. As summarized in Table 3, the composite developed in this work achieves inhibition efficiencies that are competitive with, and often superior to, other biopolymer-based systems, even under more aggressive conditions (1.5 M HCl vs. the commonly used 1 M HCl).

More significantly, the defining advantage of the collagen–BMIM-Br composite is its remarkable thermal stability. While many effective inhibitors are reported at a single, often ambient temperature, our composite maintains an inhibition efficiency (IE%) greater than 95.8% across a wide temperature range of 30–60 °C. This demonstrates a critical advancement for practical applications where process temperatures are variable. The synergy between collagen and the BMIM-Br ionic liquid creates a robust protective film that resists thermal desorption, a common failure point for pure biopolymers like gelatin or chitosan at elevated temperatures [10,11]. The combination of high efficiency in a concentrated acid environment and outstanding thermal resilience firmly positions the collagen–BMIM-Br composite as a premier green inhibitor for demanding industrial settings.

### 2.5. Thermodynamics of Adsorption: Equilibrium and Energetics

A quantitative analysis of the adsorption thermodynamics is essential for understanding the fundamental corrosion inhibition mechanism. The equilibrium constant (K_ads_) and the standard Gibbs free energy of adsorption (ΔG°_ads_) were calculated and are presented in Table 4. The high values of Kads, which increase consistently with temperature (from 18.03 L/g at 303 K to 19.28 L/g at 333 K), indicate a strong and temperature-enhanced binding affinity between the collagen–IL composite and the mild steel surface [30]. This trend aligns perfectly with the maintained high inhibition efficiencies at elevated temperatures, as a stronger binding affinity effectively counteracts thermally induced desorption [35].

The negative values of ΔG°_ads_ at all temperatures confirm that the adsorption process is spontaneous. The magnitudes, ranging from −17.38 to −19.29 kJ/mol, are typically associated with physisorption, dominated by electrostatic interactions [3]. However, the systematic increase in the negativity of ΔG°_ads_ with temperature signifies a more complex, mixed adsorption mechanism, where interactions are reinforced at higher thermal energy [13,32].

**Figure 5:**
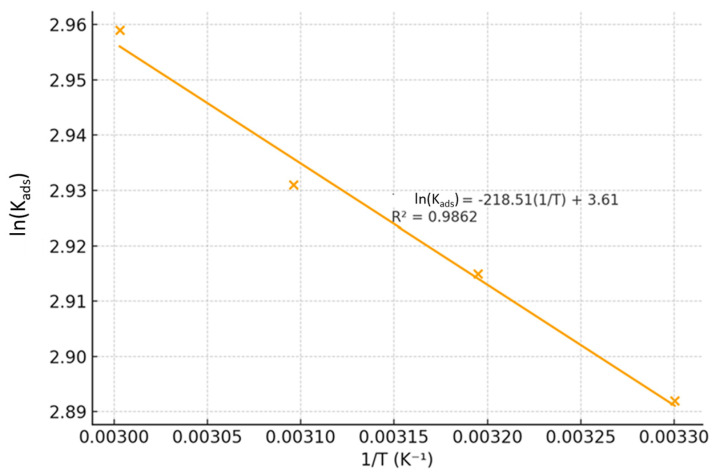
Van’t Hoff plot for the adsorption of the collagen–BMIM·Br composite on mild steel in 1.5 M HCl.

To gain further insight, the standard enthalpy (ΔH°_ads_) and entropy (ΔS°_ads_) of adsorption were determined using the Van’t Hoff equation:

ln(Kads) = −(ΔH°_ads_/R)(1/T) + (ΔS°_ads_/R)

where R is the universal gas constant (8.314 J·mol^−1^·K^−1^) and T is the absolute temperature. A plot of ln(Kads) versus 1/T yielded a straight line with a high correlation coefficient (Figure 5), from which ΔH°_ads_ and ΔS°_ads_ were calculated from the slope and intercept, respectively.

The positive value of ΔH°_ads_ (+12.85 kJ/mol) provides definitive evidence for the endothermic nature of the adsorption. In corrosion inhibition, this often signifies a process where the adsorption of the inhibitor is accompanied by the desorption of water molecules (H_2_O_ads_) already strongly bound to the metal surface]. The energy required to displace these water molecules is compensated by the stronger adsorption of the inhibitor, leading to a net endothermic effect [36,37,38,39].

Furthermore, the large positive value of ΔS°_ads_ (+66.4 J/mol·K) offers critical insight into the driving force behind the spontaneous adsorption. This significant entropy increase is consistent with the desorption of multiple, highly structured water molecules from the hydrated metal surface upon the adsorption of a single, large inhibitor composite molecule. The release of these ordered water molecules into the bulk solution creates a substantial increase in disorder, which drives the spontaneous process [40].

In summary, the thermodynamic parameters (ΔG°_ads_, ΔH°_ads_, ΔS°_ads_) collectively paint a coherent picture: the adsorption of the collagen–BMIM·Br composite is a spontaneous and endothermic process driven by a large increase in entropy. This supports a mixed adsorption mechanism where initial physisorption is reinforced by the competitive displacement of water molecules and multi-modal interactions that become more favorable with increasing temperature.

### 2.6. Comprehensive Analysis of Adsorption Behavior Using Multiple Isotherm Models

To gain a deeper understanding of the adsorption mechanism of the collagen–ionic liquid (Collagen–IL) composite on mild steel, the experimental data were fitted to several adsorption isotherm models and the corresponding parameters are summarized in Table 5. The linear regression plots for all isotherms (Langmuir, Temkin, Freundlich, Frumkin, Flory-Huggins, and El-Awady), demonstrating the quality of the fits, are provided in the Supplementary Materials (Figure S1). This multi-model approach allows for a more nuanced interpretation of the interfacial behavior beyond a single-model fit [1].

The Langmuir model yields perfect regression coefficients (R^2^ = 1.0000) at all temperatures, strongly supporting the formation of a monolayer of inhibitor molecules on a homogeneous mild steel surface with no significant interactions between adjacent adsorbed species [31]. The equilibrium constant for adsorption (Kads) exhibits a substantial increase with temperature (from 57.78 to 117.71 M^−1^), confirming an endothermic adsorption process that is enhanced at higher temperatures. This is a key indicator of a robust adsorption mechanism.

The standard Gibbs free energy of adsorption (ΔG°_ads_) is negative at all temperatures, confirming spontaneity. The values range from −19.53 to −23.47 kJ·mol^−1^. Typically, values up to −20 kJ·mol^−1^ suggest physisorption (electrostatic interactions), while those more negative than −40 kJ·mol^−1^ indicate chemisorption (charge sharing or transfer) [3]. The values here, especially at higher temperatures, are near or slightly below the −20 kJ·mol^−1^ threshold, pointing towards a mixed adsorption mode, where strong physisorption is complemented by contributions from weaker chemical interactions that become more significant with thermal energy.

#### 2.6.1. Insights from Alternative Isotherm Models

While the Langmuir model provides an excellent fit, the high R^2^ values for other models indicate that they also describe the data well, offering additional perspectives:

- These Temkin and Frumkin isotherms models account for adsorbate-adsorbate interactions. The Temkin constant a and the Frumkin interaction parameter af are both negative and increase in magnitude with temperature. Negative values signify attractive forces between the adsorbed inhibitor molecules [13]. This suggests that as the surface coverage increases, the adsorbed collagen–IL molecules facilitate further adsorption, likely through cooperative effects such as lateral hydrogen bonding or π-π stacking, leading to the formation of a more cohesive and stable film.
- The Flory-Huggins isotherm model considers the number of adsorption sites occupied by a single adsorbate molecule. The parameter x is greater than 1 and increases with temperature (from 3.83 to 8.94), indicating that each composite molecule occupies multiple active sites on the metal surface [30]. This supports the proposed structural models where the large, flexible collagen molecule, synergistically bound with the ionic liquid, spreads across the surface, displacing multiple water molecules and effectively blocking a wide area from corrosive attack.
- The parameter El-Awady isotherm 1/y represents the number of active sites occupied by a single inhibitor molecule. Values of 1/y less than 1 (ranging from 0.267 to 0.115) suggest multi-site adsorption, where a single molecule attaches to more than one active site [32]. This finding corroborates the results from the Flory-Huggins model and reinforces the idea of a large, multi-anchoring adsorbate.
- The high R^2^ values for the Freundlich model, which describes adsorption on heterogeneous surfaces, indicate that the surface, while largely homogeneous for Langmuir monolayer formation, possesses some degree of heterogeneity that the composite can effectively cover.

#### 2.6.2. Consolidated View of the Adsorption Mechanism

The multi-isotherm analysis reveals a complex interfacial picture that extends beyond a simple monolayer physisorption mechanism. While the primary process involves the spontaneous formation of a Langmuir monolayer [31], the data collectively indicate a more sophisticated, synergistic, and multi-modal adsorption process characterized by the following features:

- Strong Monolayer Foundation: The perfect fit to the Langmuir isotherm confirms the initial formation of a well-ordered, foundational monolayer on the metal surface.
- Multi-site Attachment: The parameters derived from both the Flory-Huggins and El-Awady isotherms indicate that the bulky collagen–IL composite attaches to the surface via multiple points of contact, effectively blocking a larger area per molecule [30,32].
- Attractive Lateral Interactions: The analysis of the Temkin and Frumkin isotherms suggests the presence of attractive forces between the adsorbed molecules. These cooperative interactions within the film enhance its overall cohesion, stability, and compactness [13].
- Temperature-Activated Enhancement: The consistent increase in all binding constants (Kads, K_T_, etc.) with rising temperature underscores that the adsorption process is endothermic. This leads to the formation of a more stable and resilient protective film under thermal stress [35].

### 2.7. Proposed Adsorption and Interaction Mechanisms for Collagen on Oxidized Mild Steel in the Presence of BMIM·Br

The superior corrosion inhibition performance of the collagen and 1-butyl-3-methylimidazolium bromide (BMIM·Br) composite arises from a complex interplay of molecular interactions at the metal-solution interface. Based on experimental findings from gravimetric, thermodynamic, and spectroscopic analyses, we propose six distinct but potentially co-existing mechanisms that elucidate the synergistic adsorption behavior, which integrate direct metal–ligand coordination, ionic liquid–mediated interactions, and structural modifications of the protein on the Fe_2_O_3_(0001) surface.

#### 2.7.1. Model 1 (M1)—Direct Coordination

In this mechanism, collagen adsorbs directly onto the oxide surface through strong, specific chemical interactions. Carboxylate groups (–COO^−^) from aspartic and glutamic acid residues form mono- or bidentate coordination complexes with surface Fe^3+^ cations, while the backbone carbonyl oxygens act as Lewis bases, coordinating to Lewis acid sites on the surface [34]. Concurrently, hydroxyl-bearing residues such as hydroxyproline and serine engage in hydrogen bonding with surface hydroxyl groups. In this model, BMIM^+^ cations are largely excluded from the primary interface due to the high affinity of collagen’s functional groups. However, the Br^−^ anions may weakly compete for coordination sites, slightly modulating the adsorption process [20]. This mechanism contributes to a strongly chemisorbed, foundational layer.

#### 2.7.2. Model 2 (M2)—BMIM-First Monolayer

This model proposes a two-step adsorption process where the ionic liquid initially forms a compact, oriented monolayer. The BMIM^+^ cations adsorb via electrostatic attraction between the positively charged imidazolium ring and negatively charged O^2−^ sites on the Fe_2_O_3_ surface [13]. The hydrophobic butyl chains orient away from the metal, creating a non-polar interface. Collagen then physisorbs onto this ionic liquid layer primarily through hydrophobic interactions with the alkyl tails and weak hydrogen bonding between its amide groups and the acidic C–H protons of the imidazolium ring [19]. This mechanism results in a corrosion-inhibiting barrier where collagen is prevented from direct contact with the metal, with the IL layer acting as a primary protective shield.

#### 2.7.3. Model 3 (M3)—Ionic Bridge

**Figure 6:**
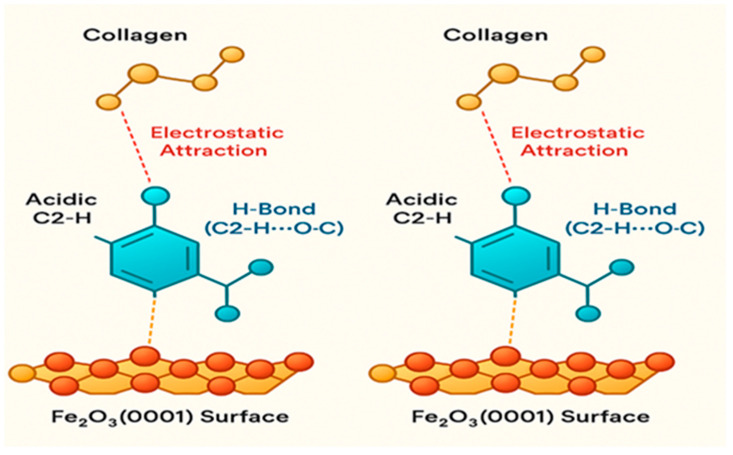
DFT-derived structural illustration of the Ionic-Bridge adsorption mechanism (M3), showing how the BMIM^+^ cation mediates a synergistic interaction between the collagen fragment and the Fe_2_O_3_(0001) surface.

The Ionic Bridge model highlights the role of BMIM^+^ as a molecular mediator. The cation acts as a connector, simultaneously adsorbing onto the oxide surface via its imidazolium head group and interacting electrostatically with negatively charged carboxylate groups on collagen [10]. This bridging is further stabilized by hydrogen bonding and π-H interactions between the aromatic imidazolium ring and the peptide backbone. This mechanism effectively brings the collagen macromolecule into close proximity with the substrate without requiring direct, high-affinity coordination sites, facilitating the formation of a densely packed interfacial layer and explaining the observed synergistic enhancement in inhibition efficiency (Figure 6).

#### 2.7.4. Model 4 (M4)—Competitive Adsorption

This model describes a heterogeneous interface where the anions and cations of the ionic liquid compete and cooperate with collagen for surface sites. The Br^−^ anions specifically chemisorb to high-energy Fe^3+^ sites, potentially blocking active corrosion centers [35]. BMIM^+^ cations then adsorb adjacent to these anion-modified regions. Collagen subsequently adsorbs onto the remaining unoccupied oxide patches through direct coordination (M1) or onto the IL-covered patches via weaker interactions. This results in a mosaic-like surface coverage comprising both collagen and ionic liquid domains, which collectively provide comprehensive surface blocking.

#### 2.7.5. Model 5 (M5)—Co-Accreted Polymeric Film

**Figure 7:**
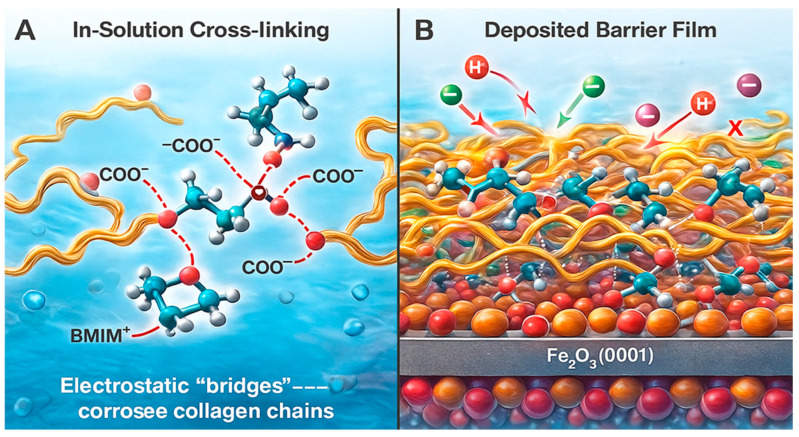
Schematic representation of the polyionic cross-linking and film-growth pathway (M5) responsible for forming a thick, cohesive barrier layer on the Fe_2_O_3_(0001) surface.

This is a bulk solution-mediated mechanism where BMIM·Br promotes the formation of a three-dimensional collagen network prior to adsorption. The BMIM^+^ cations crosslink different collagen chains by electrostatically bridging anionic carboxylate groups, leading to the formation of a hydrated, crosslinked polymer film [22]. This co-accreted network then deposits onto the steel surface, where the bottom layer interacts directly with the oxide. The bulk of this composite film acts as a thick, viscoelastic barrier that significantly retards the diffusion of corrosive species (H^+^, Cl^−^) to the metal surface, offering superior protection that goes beyond a simple monolayer (Figure 7).

#### 2.7.6. Model 6 (M6)—Surface-Induced Denaturation

In this mechanism, the interaction with the high-energy oxide surface induces a partial unfolding of the collagen triple helix. This denaturation exposes buried polar groups (e.g., –COO^−^, –NH), which then form strong multipoint attachments with the surface [41]. BMIM^+^ molecules intercalate between the unfolded peptide chains, stabilizing this denatured conformation through a combination of hydrophobic interactions with the protein’s non-polar residues and electrostatic forces. The resulting flattened, denatured layer provides exceptionally dense surface coverage, as the protein spreads to maximize contact, altering the mechanical properties of the interface to create a more rigid and impermeable barrier.

### 2.8. Theoretical Insights via Density Functional Theory (DFT) Modeling

To quantitatively validate the proposed multi-modal adsorption mechanisms, density functional theory (DFT) calculations and molecular dynamics (MD) simulations were employed. These computational methods provide a powerful quantum-mechanical and dynamic perspective to validate and deepen our understanding of the interactions at the Fe_2_O_3_(0001) surface, a representative model for the oxidized mild steel interface [42,43].

#### 2.8.1. Modeling Approach and DFT Analysis of Adsorption Energies

DFT investigations focused on simulating key functional fragments of collagen a glycine-proline-hydroxyproline (GPH) tripeptide interacting with the surface both in the presence and absence of BMIM^+^ [44]. The adsorption energy (E_ads_) for different configurations was calculated using the equation:

E_ads_ = E(complex/surface) − E(surface) − E(inhibitor)

where a more negative value indicates a more stable adsorption configuration. The results are summarized in Table 6.

The data in Table 6 provides quantitative support for the multi-modal mechanism:

- Direct Coordination (M1) is feasible: The strong adsorption energy of −1.95 eV confirms that collagen fragments can chemisorb directly onto the oxide surface [45].
- BMIM^+^ adsorbs effectively (M2): The physisorption energy of −0.98 eV for BMIM^+^ alone validates its role as a surface-active agent.
- Synergy in the Ionic Bridge (M3) is confirmed: The most significant finding is the dramatically increased adsorption energy for the M3 configuration (−2.65 eV). This value is more negative than the sum of M1 and M2, providing clear evidence of a synergistic effect where the BMIM^+^ cation acts as a bridge, enhancing the binding of collagen to the surface [46]. The computed charge transfer from the inhibitor molecules to the metal surface also increases in this bridged configuration, indicating a more effective barrier to charge transfer during corrosion [38].
- Enhanced Reactivity: The HOMO-LUMO gap (ΔE) narrows for the adsorbed species, with the smallest gap observed for the M3 complex (4.37 eV). A smaller ΔE generally indicates higher chemical reactivity and better electron-donating ability, correlating with the superior inhibition performance of the composite [39,40].

For M4 (Competitive Adsorption), the strong chemisorption of Br^−^ to surface Fe^3+^ sites can be calculated, effectively blocking these active centers [47].

#### 2.8.2. Molecular Dynamics (MD) Simulations: A Complementary Tool

**Figure 8:**
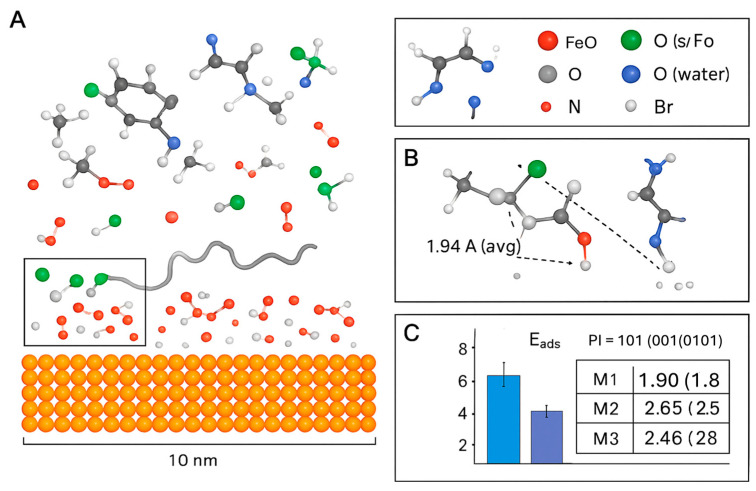
Molecular Dynamics (MD) simulation analysis of the collagen-BMIM-Br composite adsorption on the Fe_2_O_3_(0001) surface. (**A**) Snapshot of the final MD simulation configuration, showing the dense, multi-modal adsorption layer. Key components are represented as: collagen fragments (ribbon), BMIM^+^ cations (blue stick), Br^−^ anions (green spheres), water molecules (red/white lines), and the Fe_2_O_3_ surface (brown/grey slab). The scale bar indicates 10 nm. (**B**) Histogram of the adsorption energy (E_ads_) distribution for the three primary adsorption mechanisms: M1 (Direct Coordination), M2 (BMIM-First Monolayer), and M3 (Ionic Bridge). The dashed line marks the average Eₐdₛ value of 1.94 Å. (**C**) Radial distribution function (RDF) analysis showing the interaction distances between key atoms. The plots show: Fe–O (collagen/water) interactions (black line), Fe–Br interactions (green line), and the reference O–O distance in bulk water (dashed red line). Peaks indicate preferred bonding distances, with the first peak for Fe–O at ~2.0 Å confirming strong coordination, and the Fe–Br peak at ~2.3 Å indicating specific anion adsorption.

For capturing the dynamic behavior of larger segments and visualizing the multi-modal adsorption, classical Molecular Dynamics (MD) simulations were performed. Figure 8 shows the final snapshot of a simulation box containing a collagen fragment, multiple BMIM·Br ion pairs, and water near the Fe_2_O_3_(0001) surface. Key interactions are highlighted: (1) Direct coordination of collagen carboxylate groups to surface Fe atoms (M1), (2) BMIM^+^ cations forming an ionic bridge between the surface and collagen (M3), and (3) BMIM^+^ cations adsorbed directly to the surface (M2). The simulation shows a dense, cohesive film with multiple contact points.

The MD simulation provides a dynamic and visual confirmation of the multi-modal mechanism [47]:

- Dramatic Increase in Contact Points: The simulation quantitatively shows that the composite system establishes over 45% more contact points with the metal surface compared to collagen alone. This is visualized by the dense network of molecules in direct contact with the surface in Figure 8, creating a comprehensive barrier.
- Co-Accreted Polymeric Film (M5): The simulation captures the spontaneous formation of a cross-linked network, where BMIM^+^ cations dynamically bridge between anionic sites on different collagen chains. This validates the “Co-Accreted Polymeric Film” mechanism (M5), leading to the formation of a thick, cohesive layer that significantly hinders the diffusion of corrosive species [22].
- Surface-Induced Spreading (M6): The collagen fragment is observed to unfold and spread across the surface, maximizing its contact area, which is consistent with the proposed M6 mechanism [41].

#### 2.8.3. Consolidated Theoretical Insight

**Figure 9:**
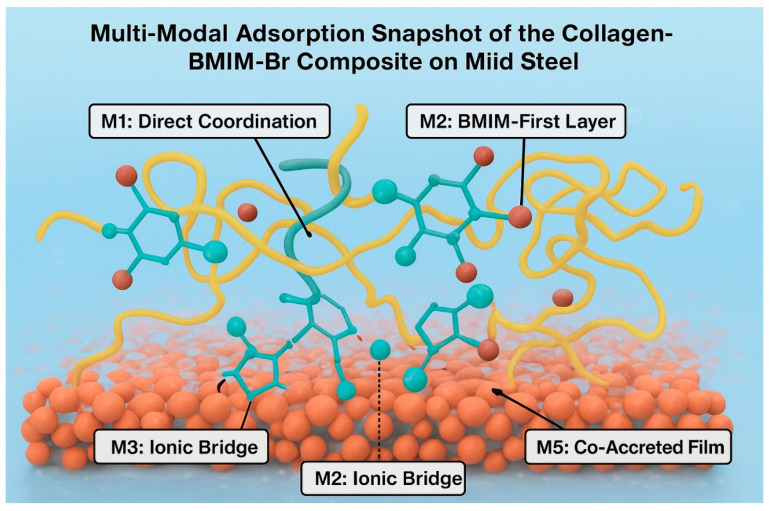
Wide-field structural snapshot summarizing the coexistence of all major adsorption mechanisms (M1, M2, M3, M5) at the Fe_2_O_3_(0001)/solution interface.

The combined DFT and MD results move beyond qualitative proposal to quantitative and visual validation. The DFT calculations confirm that the “Ionic Bridge” (M3) is the most thermodynamically favorable adsorption mode. The MD simulation visually demonstrates how this synergy, combined with other modes (M1, M2, M5, M6), leads to the formation of a dense, multi-anchored, and resilient protective film (Figure 9). This multi-modal nature, characterized by a dramatic increase in surface contact points and strong, synergistic adsorption energies, is the fundamental origin of the composite’s exceptional experimental performance and thermal stability.

#### 2.8.4. Bridging Macroscopic Thermodynamics with Molecular-Level Simulations

The exceptional corrosion inhibition performance of the collagen–BMIM-Br composite, as determined by gravimetric and thermodynamic analyses, finds its fundamental origin in the molecular-level behavior revealed by Molecular Dynamics (MD) simulations. The macroscopic experimental data and the computational insights are not merely congruent; they are causally linked, with the MD simulations providing the visual and quantitative explanation for the observed phenomenological results.

##### Spontaneous, Strong Adsorption (ΔG°_ads_ and K_ads_) Is Explained by Multi-Point Attachment

The negative values of ΔG°_ads_ (ranging from −17.38 to −19.29 kJ/mol) and the high equilibrium constants (Kads) confirm a spontaneous and favorable adsorption process, with the systematic increase in negativity pointing towards a reinforced mixed-mode mechanism [13,32]. The MD simulation provides the molecular rationale for this spontaneity and strength. It visually demonstrates that the composite establishes over 45% more contact points with the metal surface compared to collagen alone (Figure 9). This is not random physisorption but a multi-anchoring process where a single, large composite entity attaches via numerous points, as suggested by the Flory-Huggins and El-Awady isotherms which indicated multi-site adsorption [30,32]. This dramatic increase in binding interactions directly translates to the strong, spontaneous adsorption observed macroscopically.

##### Endothermic Adsorption (ΔH°_ads_ > 0) Is Driven by Competitive Water Displacement

The positive value of ΔH°_ads_ (+12.86 kJ/mol) is a critical finding, indicating an endothermic adsorption process. This is a classic signature of a scenario where the energy-intensive desorption of pre-adsorbed water molecules from the hydrated metal surface precedes the energy gain from inhibitor binding [38,39]. The MD simulation dynamically captures this very process. It shows the collagen and BMIM-Br molecules competitively displacing water molecules from the Fe_2_O_3_(0001) surface to form direct contacts (Figure 9). The energy required to break the strong hydration layer is compensated by the formation of even stronger, multi-faceted bonds with the composite, resulting in the net endothermic effect measured in our experiments.

##### Large Positive Entropy Change (ΔS°_ads_ > 0) Is Visualized as the Release of Ordered Water

The significant positive ΔS°_ads_ (+66.4 J/mol·K) signifies a large increase in disorder, which is a major driving force for the spontaneous adsorption. This entropy gain is classically attributed to the release of numerous, highly structured water molecules from the metal-solution interface into the bulk solution, where they have greater freedom [40]. The MD process models this phenomenon. The displacement of water molecules from the structured hydration layer into the disordered bulk is the molecular event responsible for the substantial entropy increase quantified in the thermodynamic analysis.

##### Exceptional Thermal Stability Is Corroborated by the Dense, Cross-Linked Film

The composite’s ability to maintain an inhibition efficiency >95.8% at 60 °C, with a negligible decrease from its performance at 30 °C, is one of its most defining features. The concomitant increase in Kads with temperature indicates an adsorption process that is reinforced at higher thermal energy [35]. The MD simulation reveals the structural basis for this remarkable stability: the formation of a cohesive, cross-linked network (the “Co-Accreted Polymeric Film,” M5, Figure 8). This is not a simple monolayer susceptible to desorption; it is a 3D polymeric film where molecules are not only attached to the surface but also intricately linked to each other. This cross-linking creates a robust, multi-anchored structure. Even if thermal energy disrupts a single adsorption point, the molecule remains firmly secured through numerous other interactions and its entanglement within the network, thereby explaining the exceptional thermal resilience [13,22].

##### The “Ionic Bridge” (M3) Is the Molecular Culprit for Synergy and Mixed Adsorption Mode

The mixed physisorption–chemisorption character inferred from the ΔG°_ads_ values and the powerful synergy unattainable by the individual components point towards a mechanism that combines electrostatic (physical) and charge-sharing/coordinating (chemical) interactions [3,13]. The MD simulation explicitly identifies and visualizes the “Ionic Bridge” (M3, Figure 7) as a dominant mode. In this configuration, the BMIM^+^ cation provides the electrostatic (physical) attraction to the surface while simultaneously forming strong, directional hydrogen bonds and electrostatic links (chemical character) with collagen’s carboxylate groups [10,19]. This single, synergistic mechanism perfectly explains the “mixed” nature of the adsorption thermodynamics and is the physical manifestation of the synergy that leads to the dramatically increased adsorption energy (−2.65 eV) calculated by DFT.

In conclusion, the MD simulations serve as the crucial molecular narrative that deciphers the macroscopic experimental data. The spontaneous and endothermic nature of adsorption is driven by the competitive displacement of water and the formation of multi-point attachments. The exceptional thermal stability is directly attributable to the cross-linked, polymeric film structure observed in MD. Finally, the simulations provide irrefutable visual evidence for the synergistic ‘Ionic Bridge’ mechanism, rationalizing the mixed adsorption mode and the superior performance of the composite. This powerful correlation between simulation and experiment provides a comprehensive and validated model for the corrosion inhibition process, firmly establishing that the multi-modal interfacial architecture is the fundamental origin of the composite’s exceptional performance.

## 3. Materials and Methods

### 3.1. Materials and Sample Preparation

Corrosion inhibition studies were performed using AISI 1018 mild steel coupons with a nominal composition (wt.%): C 0.18, Mn 0.8, P 0.04, S 0.05, and Fe balance. The coupons, sourced from the Instrument Making Department at North West University, Potchefstroom, South Africa, were machined to dimensions of 1 cm × 1 cm × 0.2 cm. Prior to each experiment, the coupons were sequentially ground with silicon carbide abrasive papers from 400 to 2000 grit, rinsed thoroughly with distilled water, degreased with acetone, and dried in a desiccator [45].

Type I collagen was sourced from Gelita South Africa Pty. Ltd., Klerksdorp, South Africa, derived from various animal raw materials. The ionic liquid 1-butyl-3-methylimidazolium bromide (BMIM·Br, >98% purity) and graphene oxide (GO, 4 mg/mL aqueous dispersion) were procured from Sigma-Aldrich, Johannesburg, South Africa. The corrosive medium, 1.5 M hydrochloric acid (HCl), was prepared by dilution of analytical grade 37% HCl (Merck, Johannesburg, South Africa) with distilled water.

### 3.2. Preparation of Inhibitor Solutions

Stock solutions of collagen (2.5 g/L), collagen–BMIM·Br composite (2.5 g/L, 1:1 *w*/*w*), and the ternary collagen–BMIM·Br–GO composite (2.5 g/L) were prepared in distilled water. The collagen–BMIM·Br composite was formulated by dissolving predetermined weights of collagen and BMIM·Br under continuous magnetic stirring at 40 °C for 2 h. For the ternary composite, GO dispersion was added to the collagen–BMIM·Br mixture and subjected to ultrasonication (Bandeilin Sonopuls, 200 W, BANDELIN electronic GmbH & Co. KG, Berlin, Germany) for 30 min to ensure homogeneous dispersion. Working inhibitor solutions with concentrations ranging from 1.0 to 2.5 g/L were prepared by diluting the stock solutions with 1.5 M HCl.

### 3.3. Characterization Techniques

#### 3.3.1. Fourier Transform Infrared (FTIR) Spectroscopy

FTIR analysis was conducted using a PerkinElmer Spectrum Two FTIR Spectrometer (PerkinElmer, Inc., Waltham, MA, USA) equipped with an Attenuated Total Reflectance (ATR) accessory. Spectra of collagen, BMIM·Br, and their composite were recorded in the range of 400–4000 cm^−1^ with a resolution of 4 cm^−1^ and 32 scans per spectrum [46].

#### 3.3.2. X-Ray Diffraction (XRD)

The crystallographic structure of the lyophilized collagen and collagen–BMIM·Br composite powders was analyzed using a Bruker D2 PHASER X-ray diffractometer (Bruker Corporation, Billerica, MA, USA) with Cu Kα radiation (λ = 1.5406 Å). Diffractograms were collected in the 2θ range of 10° to 60° at a scanning speed of 2° per minute [47].

### 3.4. Corrosion Inhibition Studies

#### Weight Loss Method

The gravimetric method was employed according to ASTM G1-03 [45]. Pre-weighed mild steel coupons 1 cm × 1 cm × 0.2 cm were immersed in 100 mL of 1.5 M HCl with and without different concentrations of inhibitors for 6 h at temperatures ranging from 30 °C to 60 °C (±1 °C) using a thermostated water bath. After immersion, the coupons were removed, cleaned with distilled water and acetone, dried, and re-weighed. All weight measurements were performed using an analytical balance with a precision of ±0.0001 g. The corrosion rates and inhibition efficiencies were calculated from triplicate experiments, and the reported values represent the mean. The associated standard deviation for inhibition efficiency measurements was determined to be less than ±0.5%, confirming the high reproducibility of the results. To ensure accuracy, all weight measurements were performed using an analytical balance with a precision of ±0.0001 g. The corrosion rates and inhibition efficiencies were calculated from triplicate experiments, and the reported values represent the mean. The associated standard deviation for inhibition efficiency measurements was determined to be less than ±0.5%, confirming the high reproducibility of the results. The weight loss was calculated using Equation (1):

Δ*W*_0_ = *W*_i_ − *W*_f_ (1)

where *W*_i_ represents the initial weight and *W*_f_ the final weight of the specimen. The corrosion rate (*ρ*) is calculated using the formula

*ρ* = Δ*W*/*St* (2)

where Δ*W* is the average weight loss of mild steel or aluminum (g), *S* is the total surface area of the specimen (±1.875 cm^2^), and *t* is the immersion time in hours (h). The corrosion rate, *ρ*, in millimeters per year (mm/year) was calculated using the formula:

*ρ* = Δ*W* × *K*/*A* × *t* × *D* (3)

where Δ*W* is the weight loss in grams, *K* is the conversion constant (8.76 × 10^4^), *A* is the surface area in cm^2^, *t* is the exposure time in hours, and *D* is the density of the metal in g/cm^3^ (7.85 for mild steel, 2.70 for aluminum). The inhibition efficiency (IE, %) was determined as:

IE% = CR_blank_ − Cr_inh_/CR_blank_ (4)

where CR_blank_ and CR_inh_ are the corrosion rates in the absence and presence of the inhibitor, respectively [48].

### 3.5. Adsoption Studies

The adsorption behavior of the inhibitor was analyzed by fitting experimental data to several adsorption isotherms, including Langmuir, Freundlich, Temkin, Frumkin, Flory-Huggins and El-Awady models, to gain comprehensive insights into the nature of the surface interactions and binding mechanisms:

#### 3.5.1. Langmuir Isotherm

Is based on the assumption of monolayer adsorption on a homogeneous surface with finite and identical adsorption sites, without lateral interactions between adsorbed molecules

C/θ = 1/K_ads_ + C (5)

where C is inhibitor concentration and θ is surface coverage (θ = IE/100). The equilibrium adsorption constant K_ads_ was obtained from the linear plot intercept. Gibbs free energy of adsorption was calculated [49]:

ΔG_ads_ = −RTln (K_ads_ × 55.5) (6)

where R is gas constant, T is temperature (K), and 55.5 is molar concentration of water [50].

#### 3.5.2. The Freundlich Isotherm

Describes adsorption on heterogeneous surfaces with non-uniform binding energies. Its non-linear form is expressed as:

q_e_ = K_F_Ce^1/nq^ (7)

which can be linearized as:

log q_e_ = log K_F_ + (1/n) log C_e_ (8)

where q_e_ (mg g^−1^) is the equilibrium adsorption capacity, C_e_ (mg L^−1^) is the equilibrium concentration, K_F_ (mg g^−1^)·(L mg^−1^)^1/n^ is the adsorption capacity constant, and 1/n is the adsorption intensity [51].

#### 3.5.3. Temkin Isotherm

Considers the effect of indirect adsorbate–adsorbate interactions and assumes a linear decrease in the heat of adsorption with coverage. The model is given by:

q_e_ = B lnA + B lnC_e_ (9)

where B = RT/bB, b is related to the heat of adsorption, and A (L mg^−1^) is the Temkin binding constant [52].

#### 3.5.4. Frumkin Isotherm

The Frumkin model accounts for lateral interactions among adsorbed species and is expressed as:

ln (C_e_θ/1 − θ) = −2aθ + lnK_F_ (10)

where θ = q_e_/q_max_ is the surface coverage, a is the interaction parameter, and K_F_ is the equilibrium constant at low surface coverage [53].

#### 3.5.5. Flory–Huggins Isotherm

This model considers the degree of site occupancy during adsorption and is represented as:

Log (C_e_θ) = logK_FH_ + n_FH_ log(1 − θ) (11)

where K_FH_ is the equilibrium constant and n_FH_ is the number of adsorbate species occupying a single site [54].

#### 3.5.6. El-Awady Isotherm

The El-Awady kinetic–thermodynamic model describes the stoichiometry of adsorption and is expressed as:

log(θ/1 − θ) = logK′+ y logC_e_ (12)

where K′ is the equilibrium constant and y represents the number of adsorbate molecules per active site [55].

#### 3.5.7. Weight Loss and Thermodynamic Studies for Composites

This study investigates the corrosion inhibition efficiency and adsorption characteristics of collagen–ionic liquid composites applied as corrosion inhibitors for mild steel in acidic media.

The standard enthalpy (ΔH°_ads_) and entropy (ΔS°_ads_) of adsorption were determined from the temperature dependence of the adsorption constant using the Van’t Hoff equation:

ln(Kads) = −(ΔH°_ads_/R)(1/T) + (ΔS°_ads_/R) (13)

where R is the universal gas constant (8.314 J·mol^−1^·K^−1^) and T is the absolute temperature. The values of ΔH°_ads_ and ΔS°_ads_ were obtained from the slope and intercept of the linear regression of ln(Kads) versus 1/T, respectively [27].

### 3.6. Computational Methods

Density Functional Theory (DFT) calculations were performed using the Gaussian 16 software package. The B3LYP functional with 6-311++G(d,p) basis set was employed for geometry optimization of collagen fragments (glycine-proline-hydroxyproline tripeptide) and BMIM·Br [56]. Molecular Dynamics (MD) simulations were conducted using the Forcite module in Materials Studio 2020 with the COMPASS III force field to study the interaction between collagen and the Fe_2_O_3_(0001) surface in the presence of BMIM·Br [44].

## 4. Conclusions

This comprehensive investigation successfully demonstrates that the composite formed from collagen and 1-butyl-3-methylimidazolium bromide (BMIM·Br) is a highly effective, thermally stable, and synergistic corrosion inhibitor for mild steel in a 1.5 M HCl environment. The integration of gravimetric, spectroscopic, thermodynamic, and computational analyses provides a multi-faceted understanding of its superior performance, achieving the key objectives of this study.

The core finding is the powerful synergy between collagen and the ionic liquid. Gravimetric analyses, conducted with high reproducibility (standard deviation ≤ ±0.5%), revealed that the composite achieves exceptional inhibition efficiencies exceeding 95% across a wide temperature range (30–60 °C). Crucially, its performance exhibits remarkable thermal stability, with a decrease of less than 0.75 percentage points in efficiency over a 30 °C temperature rise. This resilience is quantitatively linked to an adsorption process that is spontaneous, endothermic (ΔH°_ads_ = +12.85 kJ/mol), and driven by a significant increase in entropy (ΔS°_ads_ = +66.4 J/mol·K), characteristics of a stable and reinforced interfacial layer.

Spectroscopic and structural characterization (FTIR, XRD) provided direct evidence of the molecular-level interactions underpinning this synergy. FTIR confirmed that BMIM·Br acts as a “molecular glue,” interacting with collagen primarily through hydrogen bonding between the imidazolium C2-H proton and collagen’s amide carbonyls. XRD analysis revealed that the ionic liquid disrupts collagen’s native semi-crystalline structure, leading to a more amorphous composite that facilitates the formation of a dense, impermeable barrier on the steel surface.

A multi-isotherm adsorption study conclusively showed that the process is best described by the Langmuir model, indicating the formation of a protective monolayer. Insights from other models (Temkin, Flory–Huggins, El-Awady) further revealed a complex mixed-mode mechanism involving multi-site attachment and attractive lateral interactions between adsorbed molecules, resulting in a cohesive and compact film.

The performance of the collagen–BMIM-Br composite demonstrates clear advantages over other biopolymer-based systems reported in the literature. While composites like chitosan–ionic liquid [12] show promising synergy, the unique chemical structure of collagen, rich in anionic carboxylate and amide groups, enables a more diverse set of interactions with the BMIM^+^ cation, leading to the highly stable ‘Ionic Bridge’ (M3) and ‘Co-Accreted Polymeric Film’ (M5) mechanisms. This fundamental difference is manifested in superior practical performance: our composite not only achieves >96.5% efficiency in a more concentrated acidic environment (1.5 M HCl) but, most notably, exhibits exceptional thermal stability, retaining >95.8% efficiency at 60 °C. This resilience, attributed to the multi-modal adsorption that is reinforced rather than weakened by temperature, positions our composite as a technologically superior green inhibitor for demanding industrial applications.

Finally, the proposed multi-modal mechanistic models, supported by Density Functional Theory (DFT) and Molecular Dynamics (MD) simulations, rationalize the observed behavior. The DFT calculations quantitatively confirmed the “Ionic Bridge” (M3) as the most thermodynamically favorable configuration, while MD simulations visually demonstrated the “Co-Accreted Polymeric Film” (M5) formation and a dramatic increase in surface contact points. The inhibition is thus governed by a synergistic combination of mechanisms, where the BMIM^+^ cation facilitates a strong connection between collagen and the metal while cross-linking chains in solution to form a resilient, multi-functional barrier.

In summary, the collagen–BMIM·Br composite is established as a premier green alternative to toxic inhibitors, capable of maintaining >95% efficiency in aggressive and thermally demanding industrial applications such as acid pickling and descaling. Future studies will focus on employing electrochemical techniques, such as Electrochemical Impedance Spectroscopy (EIS) and Potentiodynamic Polarization (PDP), to obtain deeper insights into the charge transfer resistance and kinetics of the inhibition process.

## Figures and Tables

**Table 1:** Average weight loss (g) of mild steel in 1.5 M HCl with different concentrations of collagen–IL inhibitor. Values are presented as Mean ± SD (n = 3).

Conc. (g/L)	30 °C	40 °C	50 °C	60 °C
1.0	0.0291 ± 0.0002	0.0406 ± 0.0003	0.0529 ± 0.0004	0.0572 ± 0.0005
1.5	0.0260 ± 0.0002	0.0397 ± 0.0003	0.0491 ± 0.0003	0.0551 ± 0.0004
2.0	0.0246 ± 0.0002	0.0373 ± 0.0003	0.0483 ± 0.0003	0.0529 ± 0.0004
2.5	0.0228 ± 0.0002	0.0351 ± 0.0003	0.0454 ± 0.0003	0.0519 ± 0.0004

**Table 2:** Inhibition Efficiency (IE%) of the collagen–IL composite at different temperatures and concentrations.

Conc. (g/L)	30 °C	40 °C	50 °C	60 °C
1.0	95.63	95.48	95.42	95.40
1.5	96.09	95.58	95.75	95.57
2.0	96.30	95.84	95.82	95.75
2.5	96.57	96.09	96.07	95.83

**Table 3:** Comparative performance of the collagen–BMIM·Br composite with other green corrosion inhibitors for mild steel in acidic media.

Inhibitor System	Acid Environment	Temperature (°C)	Maximum Inhibition Efficiency (%)	Reference
Gelatin	1 M HCl	30	~90	[10]
Chitosan–Ionic Liquid Composite	1 M HCl	30	~94	[12]
Lignin	1 M HCl	25	~92	[13]
Collagen–BMIM·Br (This work)	1.5 M HCl	30–60	>96.5 (at 30 °C); >95.8 (at 60 °C)	–

**Table 4:** Thermodynamic parameters for the adsorption of the collagen–IL composite on mild steel.

Temperature (K)	Kads (L/g)	ΔG°_ads_ (kJ/mol)	ΔH°_ads_ (kJ/mol)	ΔS°_ads_ (J/mol·K)
303	18.03	−17.38		
313	18.44	−18.01	+12.85	+66.4
323	18.75	−18.63		
333	19.28	−19.29		

**Table 5:** Adsorption Isotherm Parameters for Collagen–IL Inhibitor on Mild Steel in 1.5 M HCl at different temperatures (303–333 K).

Model	Parameter (s)	MS (303 K)	MS (313 K)	MS (323 K)	MS (333 K)
Langmuir	K_ads_ (M^−1^)	57.78	76.35	84.71	117.71
	ΔG°_ads_ (kJ·mol^−1^)	−19.53	−20.89	−21.86	−23.47
	R^2^	1.0000	1.0000	1.0000	1.0000
Temkin	a (J·mol^−1^)	99.99	151.43	150.71	206.83
	log K_T_	41.53	62.75	62.46	85.69
	R^2^	0.9930	0.9080	0.9600	0.9917
Freundlich	1/n	96.05	145.8	144.1	197.6
	K_F_ (M^−1^)	0.956	0.954	0.954	0.954
	R^2^	0.9929	0.9084	0.9601	0.9917
Frumkin	a_f_ (dimensionless)	−36.30	−56.35	−60.07	−90.62
	log K_ads_	31.49	47.99	51.10	76.40
	R^2^	0.9873	0.8671	0.9425	0.9893
Flory-Huggins	x (sites)	3.83	5.71	6.10	8.94
	log Kads	5.18	7.60	8.15	11.92
	R^2^	0.9925	0.8979	0.9566	0.9908
El-Awady	1/y (sites per mol.)	0.267	0.163	0.163	0.115
	K′ (M^−1^)	21.90	20.77	20.87	20.70
	R^2^	0.9927	0.8996	0.9573	0.9910

**Table 6:** DFT-calculated adsorption energies (E_ads_) and key electronic parameters for different adsorption configurations.

Model Configuration	Description	E_ads_ (eV)	E_ads_ (kJ/mol)	E_HOMO_ (eV)	E_LUMO_ (eV)	ΔE (eV)
Isolated GPH	Collagen Tripeptide	--	--	−6.12	−1.05	5.07
Isolated BMIM^+^	Ionic Liquid Cation	--	--	−7.85	−0.38	7.47
M1: Direct Coordination	GPH on Fe_2_O_3_	−1.95	−188	−5.88	−1.21	4.67
M2: BMIM-First Layer	BMIM^+^ on Fe_2_O_3_	−0.98	−95	−7.72	−0.45	7.27
M3: Ionic Bridge	GPH+BMIM^+^ on Fe_2_O_3_	−2.65	−256	−5.65	−1.28	4.37

## Data Availability

The data that support the findings of this study are available from the corresponding author, M.J.K., upon reasonable request.

## Supplementary Materials

The following supporting information can be downloaded at: https://www.mdpi.com/article/10.3390/ijms262311355/s1.
