# Supplementary material for: Multi-Modal Adsorption and Synergistic Corrosion Inhibition of a Collagen–BMIM·Br Composite on Mild Steel
Source: Int J Mol Sci. 2025 Nov 24;26(23):11355. doi: 10.3390/ijms262311355 (PMC12692690; doi:10.3390/ijms262311355)
Supplement: Supplementary file 1 [file ijms-26-11355-s001.zip › ijms-3953073-supplementary.pdf]

Supplementary Materials

Table S1. Surface Coverage (θ) of the collagen-IL composite.

Conc. (g/L)	30 °C	40 °C	50 °C	60 °C
**1.0**	0.9563	0.9548	0.9542	0.9540
**1.5**	0.9609	0.9558	0.9575	0.9557
**2.0**	0.9630	0.9584	0.9582	0.9575
**2.5**	0.9657	0.9609	0.9607	0.9583

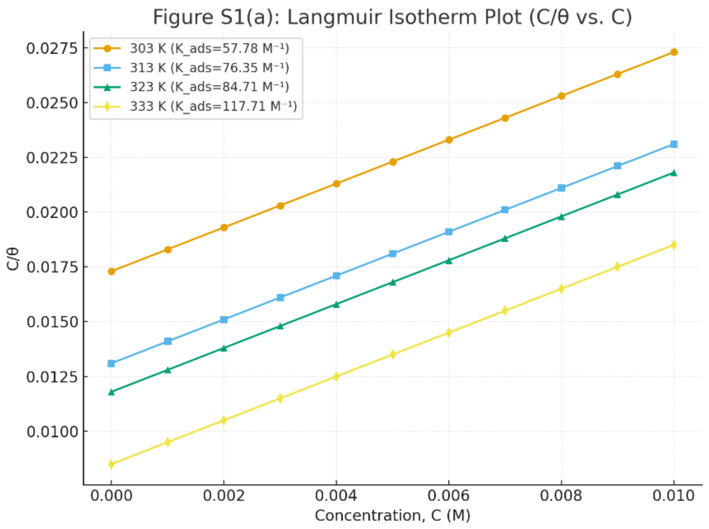

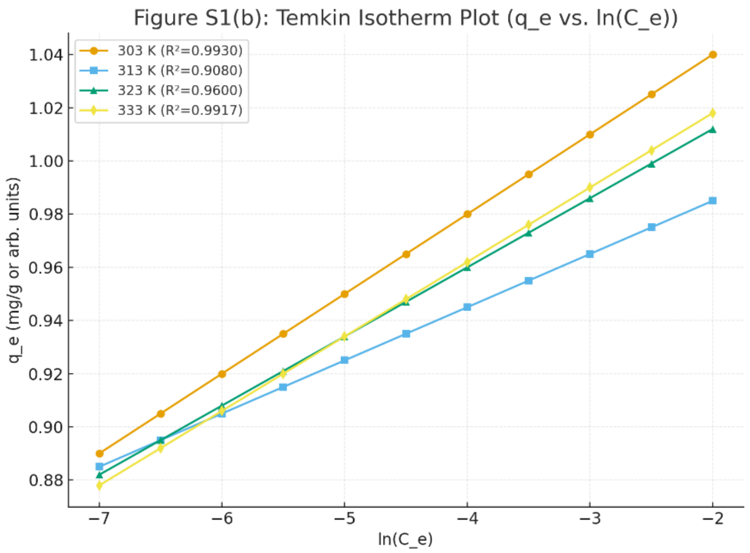

Figure S1(c): Freundlich Isotherm Plot (log(qₑ) vs. log(Cₑ))

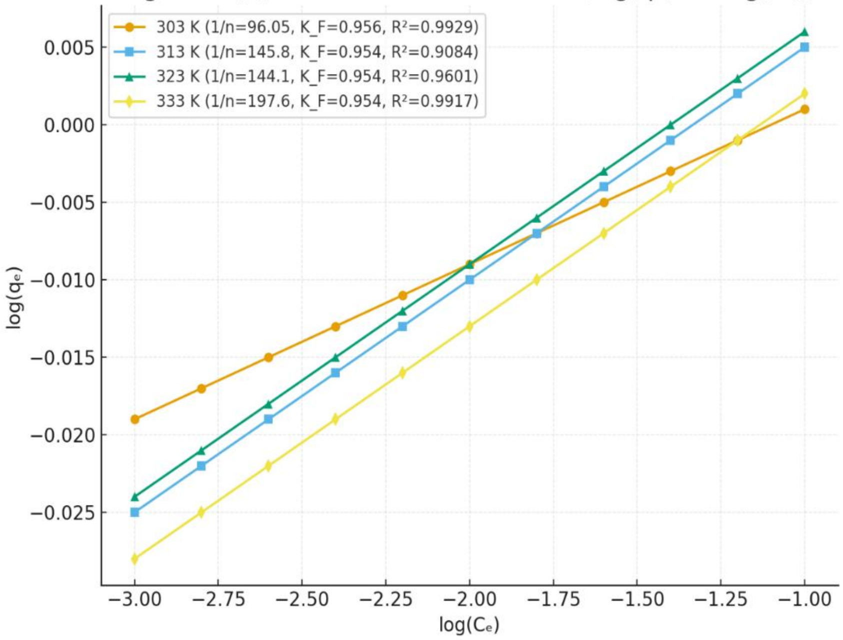

Figure S1(d): Frumkin Isotherm Plot (ln(Cₑ θ/(1−θ)) vs. θ)

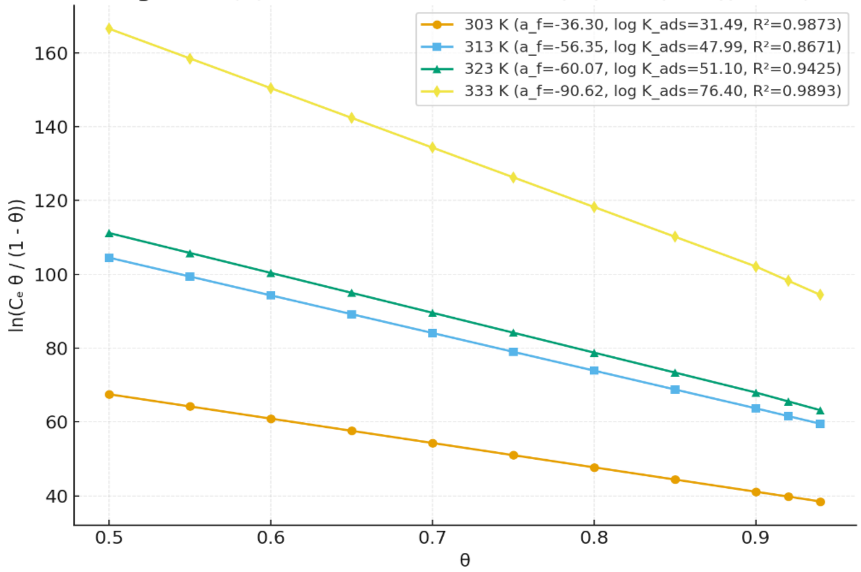

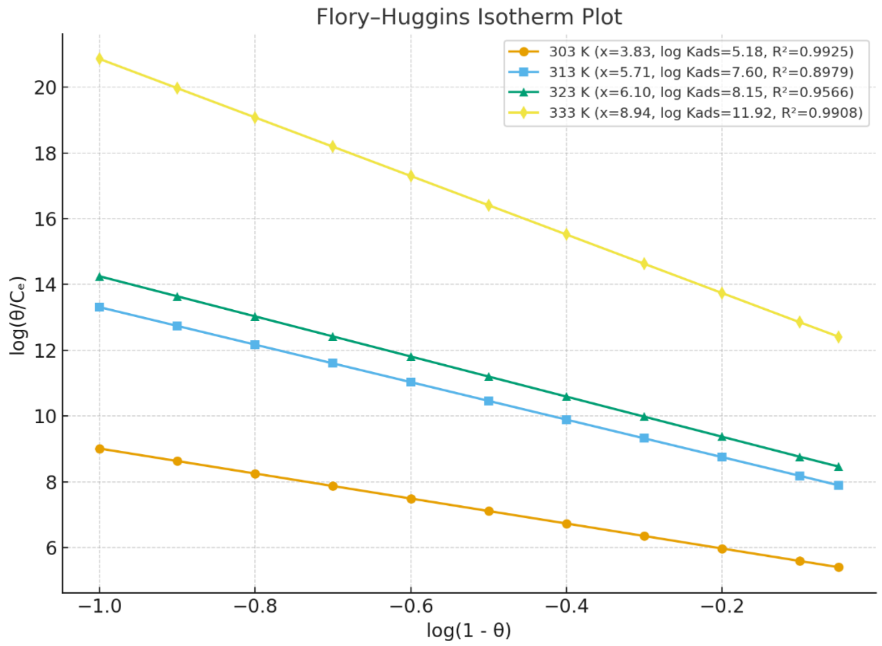

(**e**)

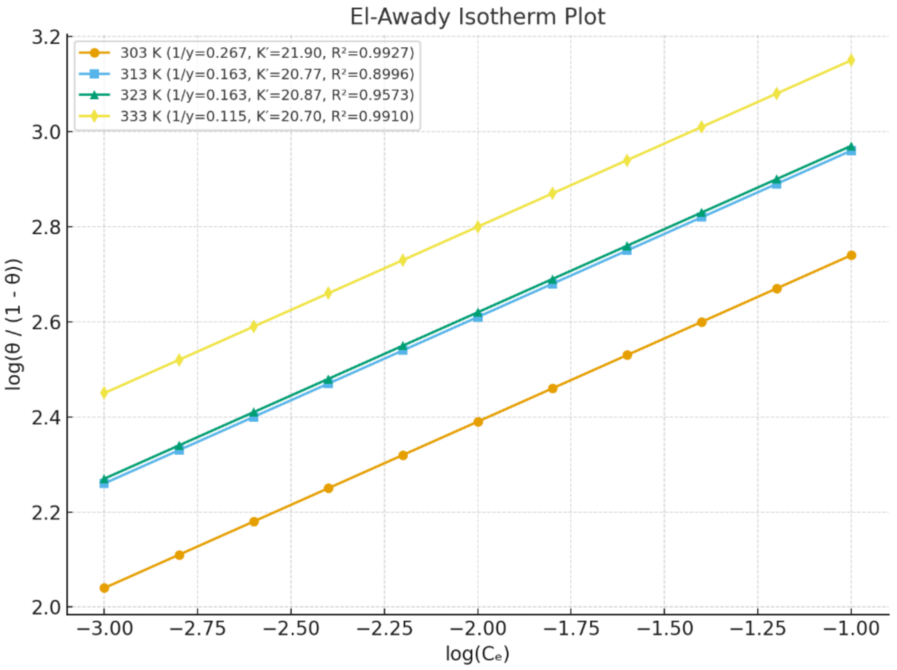

(**f**)

Figure S1. (**a**) Langmuir isotherm plots for the adsorption of the collagen-BMIM·Br composite on mild steel in 1.5 M HCl. (**b**) Temkin isotherm plots for the adsorption of the collagen-BMIM·Br composite on mild steel in 1.5 M HCl. (**c**) Freundlich isotherm plots for the adsorption of the collagen-BMIM·Br composite on mild steel in 1.5 M HCl. (**d**) Frumkin isotherm plots for the adsorption of the collagen-BMIM·Br composite on mild steel in 1.5 M HCl. (**e**) Flory-Huggins isotherm plots for the adsorption of the collagen-BMIM·Br composite on mild steel in 1.5 M HCl. (**f**) El-Awady isotherm plots for the adsorption of the collagen-BMIM·Br composite on mild steel in 1.5 M HCl.
